# Interferon-Inducible LINC02605 Promotes Antiviral Innate Responses by Strengthening IRF3 Nuclear Translocation

**DOI:** 10.3389/fimmu.2021.755512

**Published:** 2021-11-05

**Authors:** Rui Xu, Shuang-Shuang Yu, Ran-Ran Yao, Rong-Chun Tang, Jia-Wei Liang, Xuewen Pang, Jun Zhang

**Affiliations:** Department of Immunology, School of Basic Medical Sciences, NHC Key Laboratory of Medical Immunology, Ministry of Health (Peking University), Peking University Health Science Center, Beijing, China

**Keywords:** LINC02605, type I IFNs, hsa-miR-107, PTEN, IRF3, nuclear translocation

## Abstract

Non-coding RNAs represent a class of important regulators in immune response. Previously, LINC02605 was identified as a candidate regulator in innate immune response by lncRNA microarray assays. In this study, we systematically analyzed the functions and the acting mechanisms of LINC02605 in antiviral innate immune response. LINC02605 was up-regulated by RNA virus, DNA virus, and type I IFNs in NF-κB and Jak-stat dependent manner. Overexpression of LINC02605 promotes RNA virus-induced type I interferon production and inhibited viral replication. Consistently, knockdown of LINC02605 resulted in reduced antiviral immune response and increased viral replication. Mechanistically, LINC02605 released the inhibition of hsa-miR-107 on the expression of phosphatase and tensin homolog (PTEN). By microRNA mimics and inhibitors, hsa-miR-107 was demonstrated to not only inhibit PTEN’s expression but also negatively regulate the antiviral immune response. Knockdown of LINC02605 led to the reduction of PTEN expression both in mRNA and protein levels. Overexpression of LINC02605 had an opposite impact. Moreover, LINC02605 attenuated the serine 97 phosphorylation level of interferon regulatory factor 3 (IRF3) by promoting PTEN expression. Nucleoplasmic fragmentation assay showed that knocking down LINC02605 inhibited the nuclear translocation of IRF3, rendering the host cells more susceptible to viral invasion, while overexpression showed opposite effects. Therefore, LINC02605 is an induced lncRNA by viral infection and plays a positive feedback in antiviral immune response through modulating the nuclear translocation of IRF3.

## Introduction

Upon viral infection, viral nucleic acids are initially recognized by a diverse set of pattern recognition receptors (PRRs) (1). For instance, endosome TLRs (TLR3, TLR7, TLR8, TLR9) sense single-stranded RNA, double-stranded RNA, or CpG DNA respectively (2).Cytosolic RIG-I like receptors (RLRs) sense double-stranded RNA or 5’-triphosphate RNA (3). Cytosolic GMP-AMP synthase (cGAS) senses DNA (4); Nuclear hRNPA2B1 recognizes DNA (5); Nuclear IFI16 and ZBP1 recognize both RNA and DNA (6). After binding viral nucleic acids, the PRRs activate downstream signaling pathways by adaptor proteins such as MAVS or STING, then trigger the signaling cascades, eventually lead to the production of proinflammatory cytokines and type I interferons (7). The innate immune signaling pathways are fine-tuned at many aspects to ensure proper immune response is elicited.

Large-scale genomic surveys confirm that more than 80% of the human genome is involved in gene regulation, however, only <2% of the genome seems to encode proteins (8). The remaining transcription products are named as non-coding RNAs (ncRNAs). Long non-coding RNAs (lncRNAs) are a class of ncRNAs which are over 200 nucleotides in length with no protein-coding potential. There is different classification of lncRNAs. According to the direction or its relative position with the neighboring coding genes in the genome, lncRNAs can be classified into long intergenic lncRNAs (lincRNAs), natural antisense transcript (NAT), intronic transcript, bi-bidirectional lncRNAs, enhancer RNAs (eRNAs), snoRNA-ended lncRNAs (sno-lncRNAs) and so on (9, 10). LncRNAs can perform their functions by interacting with protein, RNA, DNA and acting as a decoy, scaffold, guide, sponge *etc*., to regulate gene transcription, protein translation and functions. LncRNAs represent the largest family of ncRNAs. However recently, some lncRNAs are reported to exert their functions by encoding small peptides (9). It is possible that in some instances, lncRNAs function with their peptide-encoding capacity, concurrently with or without their noncoding RNA functions.

Innate immunity is an immediate response to pathogen invasion. It induces a lot of gene transcription. LncRNAs can also be induced or down-regulated in response to viral infection (11). Given their important functions in regulating multiple cellular processes, lncRNAs are excellent candidate regulators in innate immunity. In recent years, accumulating evidences demonstrate the involvement of lncRNAs in innate immune response. For example, lincRNA-Cox2 is induced by the activation of TLR2. It can positively or negatively regulate the expression of inflammatory genes by associating with the SWI/SNF complex (12). Lnc-Lsm3b is up-regulated by VSV infection, which can compete with viral RNA to bind with RIG-I and suppress the type I IFN signaling (13). LncRNA zc3h7a, a RNA virus induced lncRNA, can promote the association of TRIM25 and RIG-I, enhance RIG-I mediated signaling (14).

Transcription factor interferon regulatory factor 3 (IRF3) plays an essential role in the induction of type I interferons (7). After the nucleic acids are detected by PRRs, IRF3 is phosphorylated, undergoes a conformational change and forms homo-dimers, then translocate to the nucleus to perform its functions (15). Kinase TBK1 or IKKϵ promotes the two-step phosphorylation of IRF3 at its C-terminus (16). The phosphorylation of IRF3 at cluster 2 (including sites Ser396, Ser398, Ser402, Thr404, and Ser405) relieves its autoinhibition and facilitates the phosphorylation of IRF3 at cluster 1 (including sites at Ser385 and Ser386) (17). The phosphorylation at cluster 1 is indispensable for IRF3 dimerization (17). Several dephosphatases can regulate the phosphorylation status of IRF3 and affect the strength of innate immunity. Protein phosphatase PP1 dephosphorylates IRF3 at Serine396 and Serine385, negatively modulating type I IFNs production (18). MAPK phosphatase 5 acts as a negative regulator in innate immune response by dephosphorylating IRF3 and impeding the formation of IRF3 dimers (19). PTEN positively regulates innate immunity by dephosphorylating IRF3 at Serine97 and promoting the nuclear translocation of IRF3 (20). To date, a lot of positive or negative regulators were reported to target at IRF3 level in innate immunity by modulating the subcellular location, protein stability or the DNA binding activity of IRF3 (15).

Regarding to the large number of lncRNAs in the human genome, only a small number of lncRNAs are reported in the regulation of the human immune system. There may be some new regulators in the innate immune response. To obtain potential novel regulators in RNA virus induced innate immune signaling, lncRNA chip was screened. In our previous study, LINC02605 is identified as a potential candidate (21). Here, we comprehensively studied the functions and mechanisms of actions of LINC02605 in innate immune signaling. Our data showed that LINC02605 is a positive regulator in innate immune response by promoting the nuclear translocation of IRF3.

## Materials and Methods

### Cell Lines

Human embryonic kidney (HEK) cell lines HEK293, HEK293T; human leukemia monocytic cell line THP-1; human cervical adenocarcinoma cell line Hela; African green monkey kidney cell line Vero and murine fibroblast cell line L929 cells were maintained in high-glucose Dulbecco’s Modified Eagle’s medium (DMEM; Thermo-Fisher Scientific, Waltham, MA, USA) supplemented with 10% heat-inactivated Fetal Bovine Serum (FSP500, Excell Bio, Shanghai, China). 100 U/ml penicillin and 100 μg/ml streptomycin (Thermo-Fisher Scientific, Waltham, MA, USA).

### Reagents

Antibody against IRF-3 was from Cell Signaling Technology (Danvers, MA, USA). Anti-PTEN antibody was from Abmart (Shanghai, China). Anti-green fluorescent protein (GFP) tag antibody was from Proteintech (Rosemont, IL, USA). Antibodies against lamin-B1 and glyceraldehyde-3-phosphate dehydrogenase (GAPDH) were from Bioworld Technology (Visalia, CA, USA). Anti-PCNA antibody was from Huabio (Hangzhou, Zhejiang Province, China). Anti-mouse and rabbit IgG-HRP antibodies were from Biodragon (Beijing, China). Antibody against phospho-IRF-3 (Ser97) was a gift from Dr. Deyin Guo (Sun Yat-sen University, China). The viral analogues polyinosinic-polycytidylic [Poly (I:C)] acid and poly (deoxyadenylic-deoxythymidylic) [Poly (dA:dT)] acid sodium salt were from *In vivo* Gen (San Diego, CA, USA). The inhibitor of STAT1 signaling Fludarabine (1.5 μM) and potent IKK2 inhibitor SC-514 (100 μM) were from Selleck (Houston, Texas, USA).

### Virus

Sendai virus (SeV) was propagated and amplified by inoculating chick embryo allantoic cavity. Herpes simplex virus-1 (HSV-1), Vesicular stomatitis virus (VSV), VSV-GFP were propagated and amplified by infection of a monolayer of Vero cells. The viral titers of SeV, HSV-1 were determined by TCID50 on L929 cells, and the viral titers of VSV and VSV-GFP were determined by plaque forming assay using HEK293 cells. Cells were infected with SeV (MOI=5), HSV-1 (MOI=5), VSV or VSV-GFP (MOI=1) for the indicated hours.

### Cloning of Full-Length LINC02605

The full-length sequence of human LINC02605 was amplified from HEK293T cells and cloned into the restriction enzyme cutting site KpnI and NotI of pcDNA3.1(+) eukaryotic expression vector by standard molecular biology techniques and confirmed by DNA sequencing. Primers used for cloning were as follows and designed based on the sequence information from NCBI: (forward) 5’-CGGGGTACCAGCTTCAACTCTGTGAAATAGGG-3’, (reverse) 5’-AATGCGGCCGCAGGAGAAAAAGCCAAGTCAGT-3’. Plasmids were transiently transfected into cells using Lipofectamine 20000 (Thermo-Fisher), or poly ethylene imine (PEI; Polyscience, IL, USA) according to the manufacturer’s instructions.

### Reverse-Transcription PCR and Quantitative Real-Time PCR

Total RNAs were extracted from fresh culture cells using TRIzol Reagent (CWBIO, Beijing, China), and subsequently were reverse transcribed into cDNA by a Reverse Transcription kit (Yeasen, Shanghai, China). Quantitative real-time PCR (qPCR) was performed with the Eco™ PCRmax system, using a qPCR SYBR Green mix (Yeasen). The primers used in this study were as follows: LINC02605#1 (for basic qPCR), (forward) 5’-GCTGCCTTGTTGGATGGGTGCT-3’, (reverse) 5’-GCATTGCCTGCTTCCGCTCT-3’; LINC02605#2 (for qPCR), (forward) 5’-CATGAACCCTGTGGGCCTTT-3’, (reverse) 5’-GCTGTTCCATCTGGGACCTA-3’; human *IFNB1*, (forward) 5’-ACTGCCTCAAGGACAGGATG-3’, (reverse) 5’-GGCCTTCAGGTAATGCAGAA-3’; VSV RNA, (forward) 5’-ACGGCGTACTTCCAGATGG-3’, (reverse) 5’-CTCGGTTCAAGATCCAGGT-3’; human *GAPDH*, (forward) 5’-ACCCACTCCTCCACCTTTGA-3’, (reverse) 5’-CTGTTGCTGTAGCCAAATTCGT-3’, and *U6*, (forward) 5’-CTCGCTTCGGCAGCACA-3’, (reverse) 5’-AACGCTTCACGAATTTGCGT-3’; human *CXCL10*, (forward) 5’-GTGGCATTCAAGGAGTACCTC-3’, (reverse) 5’-TGATGGCCTTCGATTCTGGATT-3’; human IFN-stimulated gene (*ISG*)-15, (forward) 5’-CGCAGATCACCCAGAAGATCG-3’, (reverse) 5’-TTCGTCGCATTTGTCCACCA-3’; human *PTEN* (for basic PCR), (forward) 5’-CTAGAGGAGACCCAAGGGCT-3’, (reverse) 5’-AAAAAGGGGGCCAAACTCCA-3’; human *PTEN* (for q-PCR), (forward) 5’-TGGATTCGACTTAGACTTGACCT-3’, (reverse) 5’-GGTGGGTTATGGTCTTCAAAAGG-3’.

### Small RNA-Mediated Interference

Small interference RNAs (siRNAs) were transfected into the cells by jetPRIME Transfection Reagent (Polyplus Transfection, Illkirch, France) according to the manufacturer’s instructions. The sequence of siRNAs against LINC02605 were as follows: si#1, (sense) 5’-CCUGCAAUAGCAUCUUCUUTT-3’, (antisense) 5’-AAGAAGAUGCUAUUGCAGGTT-3’; si#2, (sense) 5’-CCUAUUUCUUACCAUCCUUTT-3’, (antisense) 5’-AAGGAUGGUAAGAAAUAGGTTA-3’. A non-targeting siRNA was used as a negative control (N.C.): (sense) 5’-UUCUCCGAACGUGUCACGUTT-3’, (antisense) 5’-ACGUGACACGUUCGGAGAATT-3’.

### Transfection of Micro RNA Mimics or Inhibitor

The mimics or inhibitor of micro RNA hsa-miR-107 is transfected into the cells by jetPRIME Transfection Reagent (Polyplus Transfection) with standard procedures. The sequences are as follows: hsa-miR-107 mimics, (sense) 5’-AGCAGCAUUGUACAGGGCUAUCA-3’, (antisense) 5’-AUAGCCCUGUACAAUGCUGCUUU-3’; the N.C. of mimics, (sense) 5’-UUCUCCGAACGUGUCACGUTT-3’, (antisense) 5’-ACGUGACACGUUCGGAGAATT-3’; hsa-miR-107 inhibitor, (sense) 5’-UGAUAGCCCUGUACAAUGCUGCU-3’; the N.C. of inhibitor, (sense) 5’-CAGUACUUUUGUGUAGUACAA-3’.

### Cell Fraction Isolation and Western Blot

Cell fractions were isolated for RNA and protein analysis. For RNA, we used PARIS™ Kit (Thermo-Fisher Scientific) to extract nuclear and cytoplasmic RNA respectively, according to the manufacturer’s instructions. The expression level of target genes in two fractions was normalized with input RNA, which was set to 100%. For western blotting analysis, we gathered cells from culture plates and washed them twice with PBS. Then, suspend cells gently in EBC1 lysis buffer (50 mM Tris-HCl, 100 mM NaCl, 0.05% NP-40, 1 mM EDTA, 1 mM DTT). Incubate cells for 5 minutes on ice and then centrifuge them 500-1000 g, 5 minutes at 4°C. The supernatant was cytoplasm, and the precipitate was washed three times with EBC1 lysis buffer, then lysed 10 minutes with NTEN lysis buffer (1 M Tris-HCl, 100 mM NaCl, 0.5% NP-40, 0.5 M EDTA) to harvest nucleus. Afterwards, protein samples were separated by SDS-PAGE (sodium dodecyl sulfate-polyacrylamide gel electrophoresis) and then electransferred to a nitrocellulose membrane. Next, the samples were blocked in 5% skimmed milk for 1 hour and then incubated in the corresponding primary antibody at 4°C, overnight. By the next day, they were incubated with the corresponding secondary antibody at room temperature for 1 hour. The bands were visualized by an ECL chemiluminescence detection system (GE ImageQuant LAS 500, Massachusetts, USA).

### Dual-Luciferase Reporter Assays

HEK293T cells (2.5×10^5^/ml) were incubated on 24-well plates for 24 hours and transfected with 100 ng IFNβ-luc or ISRE-luc luciferase reporter plasmid, with 1 ng pRL-SV40 plasmid and the described gene constructs. In the next day, cells are stimulated by viruses or virus analogues for the indicated time period. Then cells were harvested and the lysate were measured with a Dual-luciferase reporter Assay System (Promega, Madison, WI, USA) according to the manufacturer’s instructions. The luciferase activity was normalized to Renilla luciferase signal. The reporter assays were performed in duplicate and repeated at least three times.

### Plaque Forming Assay

Incubate HEK293 cells (2.5×10^5^/ml) on a 24-wells plate for 24 hours beforehand. Infect THP-1 cells (2.5×10^5^/ml) with VSV virus (MOI=1) and harvest the THP-1 cells’ culture supernatant at 24 hpi. Dilute the supernatant and add them into monolayers of HEK293 cells in sequence. Incubate the cells for 1 hour, shake the plate slowly at an interval of 20 min to make the virus adsorb HEK293 cells evenly. Next, discard the virus diluent and add methylcellulose (4000cp, 2%) medium to each wells, culturing for 3 days. After that, outwell the medium and add cold paraformaldehyde (4%) to the wells, fixing the cells at room temperature for 20 min. Then, discard the content and add crystal violet solution (0.5-1%) to stain the cells. Finally, the virus titers could be obtained by counting the plague forming units (PFUs).

### Statistical Analysis

The statistical significance of comparisons between two groups were determined by unpaired Student’s *t*-test. For groups more than two, we used one-way ANOVA. The statistic difference between groups were considered at *P* < 0.05. The charts were generated and analyzed by GraphPad Prism 8.0.

## Results

### LINC02605 Is Induced by Virus Infection and Type I IFN Stimulation

Previously, in order to screen potential novel regulators in RNA virus-induced signaling pathway, by microarray analysis, LINC02605 is identified as a candidate regulator that may be involved in modulating antiviral innate immune response (21). In this study, we aimed to systematically investigate the functions and possible mechanisms of LINC02605 in antiviral innate immune response. First, we analyzed the basic features of LINC02605 by bioinformatics. LINC02605 has a long stem of double-stranded RNA and multiple single-stranded RNA loops (**Supplementary Information, Figure S1A**). Expression pattern analysis showed that LINC02605 is widely distributed in many tissues of human body (**Supplementary Information, Figure S1B**). Then the coding potential of LINC02605 is analyzed. According to ORFfinder (NCBI), LINC02605 has the longest ORF less than 300nt; lnCAR analysis showed the CPAT score < 0.364 and the CNCI score < 0 (**Supplementary Information, Figures S1C, D**). Moreover, Public internal ribosome entry sites (IRESs) database (22) revealed that LINC02605 has no ribosome occupancy (**Supplementary Information, Figure S1E**). These bioinformatics analysis suggest that LINC02605 lacks coding potential and may function as a noncoding RNA.

**Figure 1:**
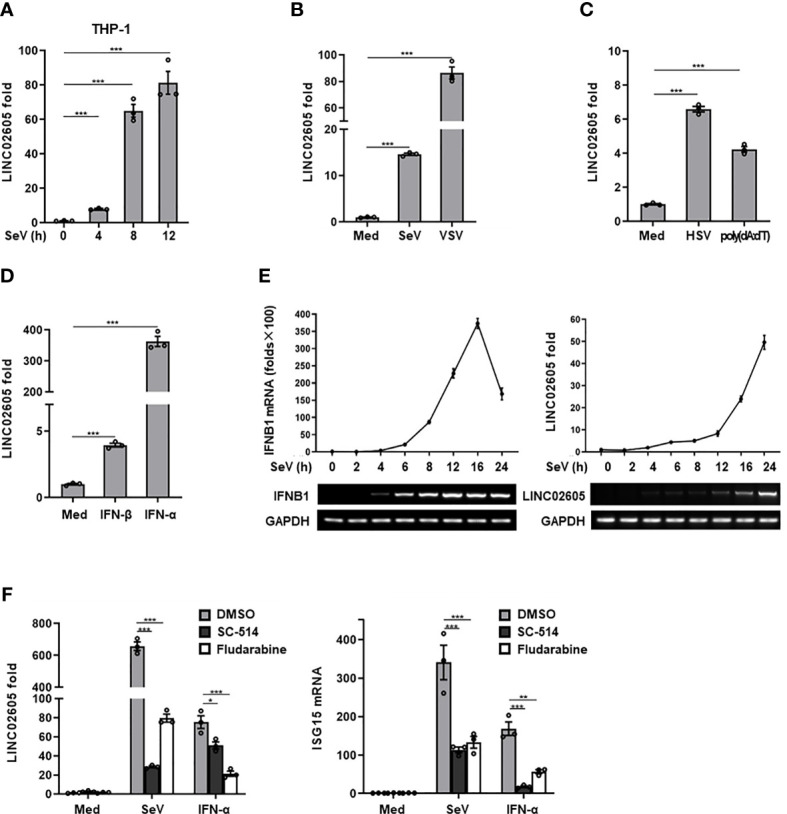
LINC02605 is upregulated by viral infection or type I IFN stimulation. **(A)** Q-PCR analysis of LINC02605 expression in THP-1 cells infected with SeV for the indicated hours. **(B–D)** Q-PCR analysis of LINC02605 expression in HEK293T cells infected with RNA virus (SeV, VSV) **(B)**, DNA virus (HSV) for 12h, stimulated with DNA virus analogue poly (dA:dT) for 12h **(C)**, or I-IFNs for 12h **(D)**. **(E)** Q-PCR analysis of *IFNB1* and LINC02605 mRNA expression in HEK293T cells infected with SeV for the indicated times. **(F)** Q-PCR analysis of LINC02605 and *ISG15* expression in HEK293T cells pretreated for 1h with the IKK-2 inhibitor SC-514 (100 μM) or the STAT1 inhibitor Fludarabine (1.5 μM), then treated with SeV or IFN-α for 12h. All data shown are from one representative experiment of at least three independent repeats with similar results. *P < 0.05, **P < 0.01 and ***P < 0.001 (Student’s t-test or ANOVA).

Then the expression pattern of LINC02605 was detected upon virus infection. Consistent with the microarray data and our previous studies (21), LINC02605 was induced by RNA virus (Sendai virus) in THP1 cells (**Figure 1A**), Hela cells (**Supplementary Information, Figure S2A**) and HEK293T cells (**Figure 1B**). Besides, LINC02605 was also up-regulated by another RNA virus (VSV) (**Figure 1B**), DNA virus HSV-1 and DNA virus mimics poly (dA:dT) (**Figure 1C**). Next, we monitored the expression level of LINC02605 by type I IFN stimulation. As shown in **Figure 1D**, LINC02605 was induced by IFN-α and IFN-β treatment. We then dynamically examined the expression level of type I IFN and LINC02605after virus infection and found that the up-regulation of LINC02605 is behind the up-regulation of IFN-β, supporting the notion LINC02065 is an interferon stimulated lncRNA (**Figure 1E**).In order to further reveal the possible signaling pathways that induce the expression of LINC02605, we analyzed the transcription factors which have the potential to bind LINC02605 promoter region (**Supplementary Information, Figure S1F**), suggesting that NF-κB and STAT are potential binding transcription factors. Then we used IKK inhibitor SC-514 and STAT inhibitor Fludarabine to detect whether NF-κB or Stat signaling pathways are involved in the induced expression of LINC02605. As shown in **Figure 1F**, the induced expression of LINC02605 by SeV or IFN-α treatment is inhibited by IKK inhibitor or Stat inhibitor. Another important aspect for a lncRNA is its localization. It is suggestive for its function. Thus, we also examined the localization of LINC02605 upon viral infection, as shown in **Supplementary Information, Figure S2B**, LINC02605 was mainly localized in the nucleus. In summary, LINC02605 is a virus or type I IFN induced gene that may be involved in antiviral immune response.

### LINC02605 Enhances Type I IFN Signaling Response to Viral Infection

**Figure 2:**
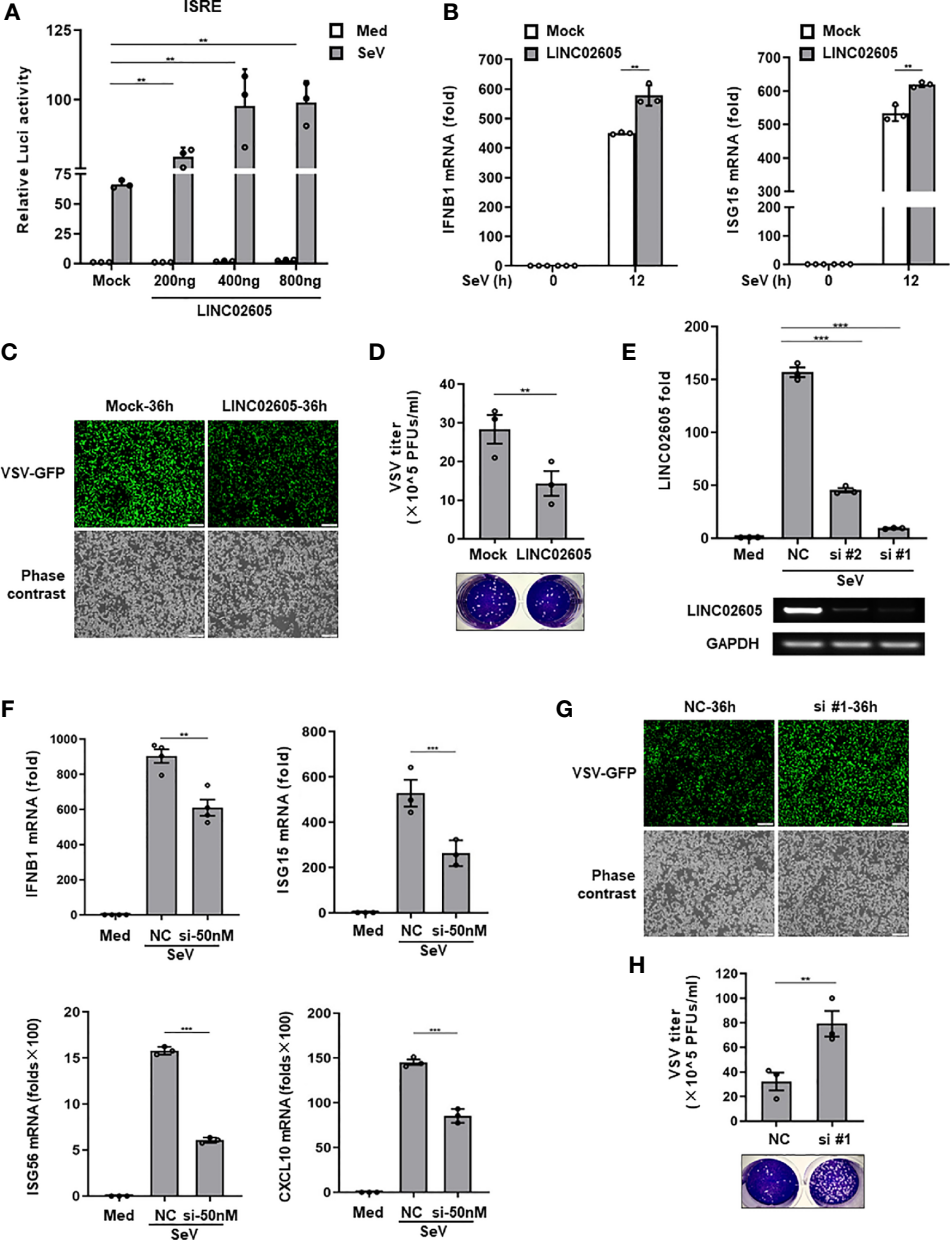
LINC02605 enhances I-IFNs production in response to viral infection. **(A)** Dual luciferase analysis of ISRE promoter activation in HEK293T cells, after co-transfecting plasmids expressing LINC02605 with indicated does (or empty vector control), an ISRE reporter and pRL-SV40 for 24h, then infected with SeV for 12h. **(B)** Q-PCR analysis of *IFNB1* and *ISG15* mRNA expression in LINC02605 expressing vector or empty vector control (mock) transfected HEK293T cells upon SeV infection for 12h. **(C)** Fluorescence analysis of VSV replication in HEK293T cells transfected by LINC02605 or mock vector which were infected with VSV-GFP for the indicated hours. Scale bar, 1mm. **(D)** THP-1 cells expressing LINC02605 or its control group were infected with VSV, and the virus titers in culture supernatants were determined through plaque forming assay. **(E)** Q-PCR analysis of LINC02605 silencing efficiency by two individual siRNA oligos (#1, #2). NC, a non-targeting control siRNA. **(F)** Q-PCR analysis of *IFNB1*, *ISG15*, *ISG56* and *CXCL10* mRNA in control or LINC02605-silenced HEK293T cells infected with SeV for 12h. **(G)** Fluorescence analysis of VSV replication in NC- or LINC02605-silenced HEK293T cells infected with VSV-GFP for the indicated hours. Scale bar, 1mm. **(H)** LINC02605-silenced THP-1 cells or the control group were infected with VSV, and the virus titers in culture supernatants were determined by plaque forming assay. All data shown are from one representative experiment of at least three independent repeats with similar results. **P < 0.01 and ***P < 0.001 (Student’s t-test or ANOVA).

Next, to unveil whether LINC02605 is indeed involved in the regulation of antiviral immune response. We constructed the full-length of LINC02605 into pcDNA3.1(+) vector (**Supplementary Information, Figure S2D**). When overexpression of LINC02605, it promotes SeV induced ISRE activation in a dose dependent manner (**Figure 2A**). The downstream genes such as *IFNB1、ISG15* were also enhanced upon SeV infection (**Figure 2B**). Consistently, the replication of RNA virus VSV was inhibited when overexpression of LINC02605 in HEK293T cells and THP1 cells (**Figures 2C, D**). To further analyze the functions of LINC02605 in antiviral immune response, endogenous LINC02605 were knock-downed by siRNA oligos in HEK293T cells (**Figure 2E**). When knockdown of LINC02605, the transcription of *IFNB1、ISG15、ISG56* upon SeV infection was reduced (**Figure 2F**). In agreement with that, the viral replication was enhanced in HEK293T cells and THP1 cells (**Figures 2G, H**). Taken together, LINC02605 is a positive regulator in antiviral innate immune response.

### Hsa-miR-107 Is a Negative Regulator in Antiviral Innate Immune Response and Inhibits the Expression of PTEN

**Figure 3:**
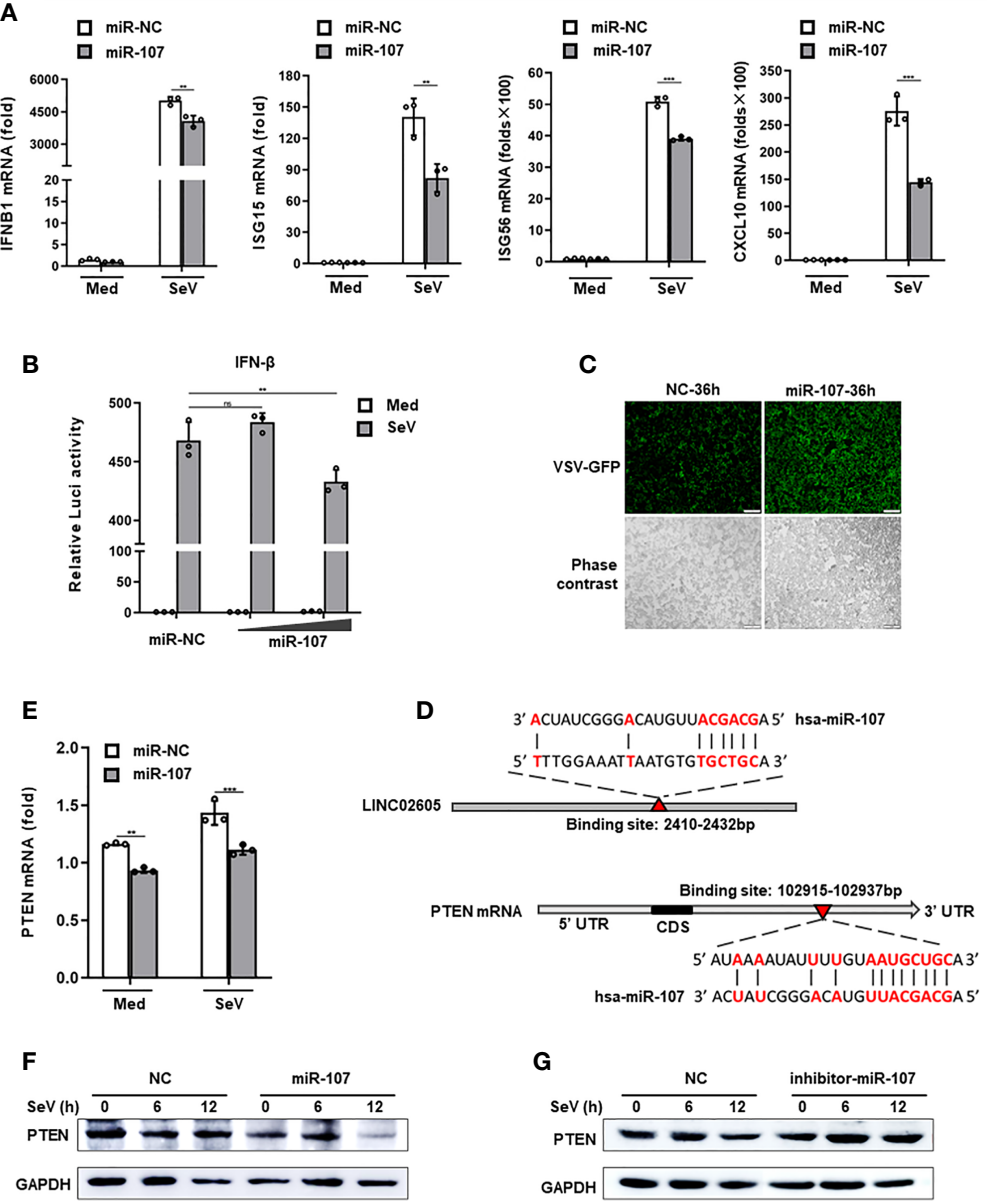
Hsa-miR-107 can negatively regulate antiviral innate immune response and inhibit the expression of PTEN. **(A)** Q-PCR analysis of *IFNB1*, *ISG15*, *ISG56* and *CXCL10* in hsa-miR-107 mimics or miR-NC transfected HEK293T cells upon infection with SeV for 12h. **(B)** Dual luciferase analysis of the IFN-β promoter activation in HEK293T cells, after co-transfecting hsa-miR-107 mimics with increasing doses (80 nmol/L, 120 nmol/L), or miR-NC, an IFN-β reporter and pRL-SV40 for 24h, then infected with SeV for 12h. **(C)** Fluorescence analysis of VSV replication in miR-NC or hsa-miR-107 mimics transfected HEK293T cells upon VSV-GFP infection for the indicated hours. **(D)** The binding sites of hsa-miR-107 on LINC02605 and PTEN were predicted by TargetScan. **(E, F)** Q-PCR and western blot analysis of PTEN expression in hsa-miR-107 mimics or miR-NC transfected HEK293T cells infected with SeV for the indicated times. **(G)** Western blot analysis of PTEN expression in hsa-miR-107 inhibitor or NC transfected HEK293T cells infected with SeV for the indicated hours. All data shown are from one representative experiment of at least three independent repeats with similar results. **P < 0.01 and ***P < 0.001; ns, no significance (Student’s t-test or ANOVA).

CeRNA is an important acting mechanism of lncRNA (23). Through bioinformatics we analyzed the miRNAs with potential binding targets of LINC02605, which were shown in **Supplementary Information, Figure S2C**). Among them, hsa-miR-107 attracts our attention due to it has subtle relationship with LINC02605 according to the published data. It is reported that LINC02605 can sponge hsa-miR-107, then regulating the expression of PTEN and participating in the development of bladder cancer (24). However, until now, the functions of hsa-miR-107 in the regulation of immune response has not yet been studied. By adding miR-107 mimics, it resulted in reduced expression of downstream genes such as *IFNB1, ISG15, ISG56* and *CXCL10* in SeV infected cells (**Figure 3A**). Luciferase reporter assay showed that miR-107 inhibited SeV induced IFN-β activation (**Figure 3B**). Consistently, RNA virus replication was enhanced in miR-107 mimics group (**Figure 3C**).

In the 3’ UTR of PTEN, it indeed harbors a miR-107 binding site (**Figure 3D**). Then we detected the effects of miR-107 on the expression of PTEN. MiR-107 mimics can down-regulate the expression of PTEN both in mRNA and protein level (**Figures 3E, F**). miR-107 inhibitor can reverse the effects of miR-107 on the expression of PTEN (**Figure 3G**; **Supplementary Information, Figure S2E**). All these data suggest that has-miR-107 negatively modulates antiviral innate immune response and the expression level of PTEN.

### LINC02605 Targets hsa-miR-107 to Enhance the Expression of PTEN

**Figure 4:**
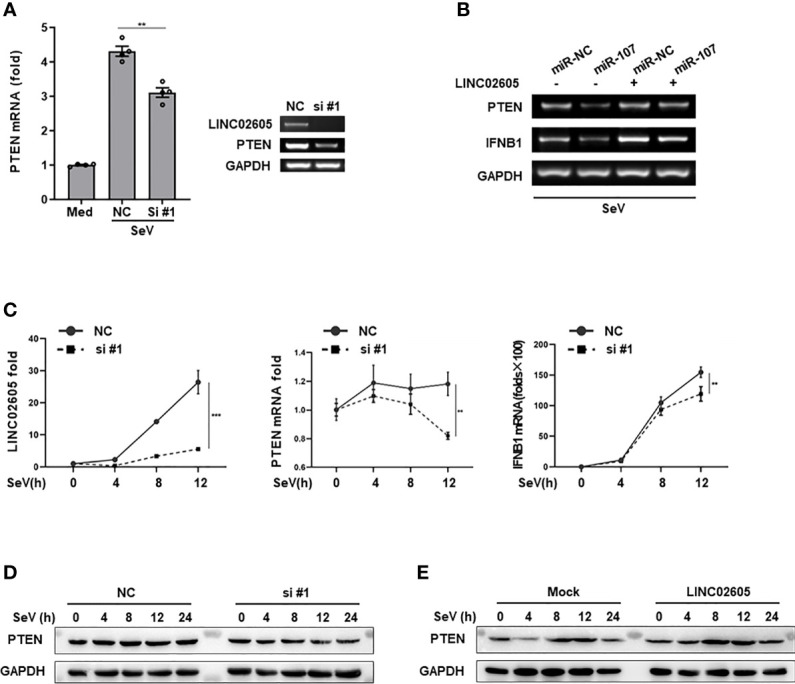
LINC02605 targets hsa-miR-107 and enhances the expressions of PTEN **(A)** Q-PCR (left) and basic PCR (right) analysis of PTEN in control or LINC02605-silenced HEK293T cells infected with SeV for 12h. **(B)** of the mRNA expression levels of *PTEN* and *IFNB1* were detected in miR-NC or hsa-miR-107 transfected WT HEK293T cells or LINC02605-overexpressing HEK293T cells infected with SeV for the indicated hours. **(C)** Q-PCR analysis of *PTEN* and *IFNB1* mRNA expression in NC- or LINC02605-silenced HEK293T cells infected with SeV for the indicated times. **(D, E)** Western blot analysis of PTEN expression in LINC02605-silenced or -overexpressed HEK293T cells upon SeV infection for the indicated hours. All data shown are from one representative experiment of at least three independent repeats with similar results. **P < 0.01 and ***P < 0.001 (Student’s t-test or ANOVA).

As mentioned above, LINC02605 has many potential binding microRNAs. Hsa-miR-107 is one of them, which can negatively regulate antiviral immune response and downregulate the expression of PTEN. Next, we tried to answer whether LINC02605 has the effects on the expression of PTEN. By knocking down of LINC02605, the mRNA expression of PTEN was reduced (**Figure 4A**). Consistent with the above results, miR-107 can down-regulate the expression level of PTEN, leading to reduced expression of *IFNB1* (**Figure 4B**). In miR-107 group, overexpression of LINC02605 can reverse the negative regulatory effects of miR-107 on the expression of PTEN, then leading to comparable expression of *IFNB1* with control group (**Figure 4B**). By overexpression or knockdown of LINC02605, the mRNA and protein level of PTEN was also detected. Knockdown of LINC02606 resulted in reduced expression of PTEN in both mRNA and protein level upon viral infection (**Figures 4C, D**). Whereas, over-expressed LINC02605 enhanced the expression of PTEN at protein level (**Figure 4E**). Thus, LINC02605 may bind to hsa-miR-107 to release the inhibitory effects of miR-107 on the expression of PTEN.

### LINC02605 Dampens the Phosphorylation Level of IRF3 at Ser97 *via* PTEN, Then Promoting the Import Of IRF3 Into the Nucleus

**Figure 5:**
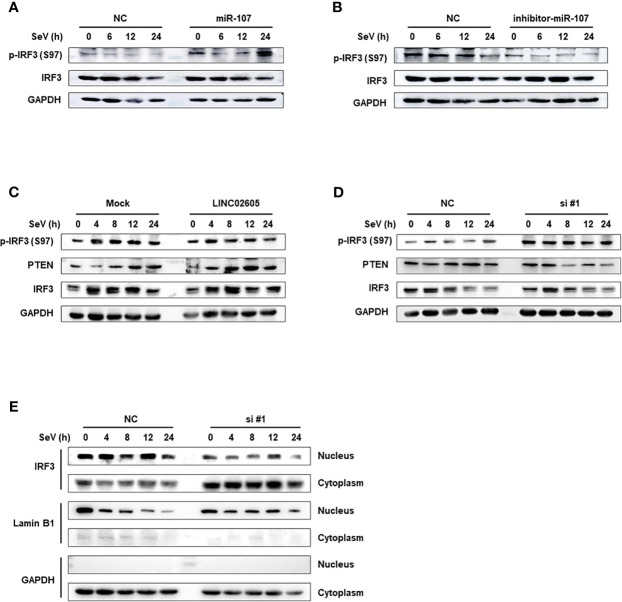
LINC02605 attenuates the phosphorylation level of IRF3 at Ser97 *via* PTEN and controls the import of IRF3 into the nucleus. **(A, B)** Western blot analysis of p-IRF3 (S97) and total IRF3 proteins in lysates of hsa-miR-107 mimics **(A)** or inhibitor **(B)** transfected HEK293T cells infected with SeV for the indicated hours. **(C)** Western blot analysis of p-IRF3 (S97) or total IRF3 in lysates of HEK293T cells transfected by ectopically expressed LINC02605 or empty vector with SeV infection for the indicated hours. **(D)** Western blot analysis of p-IRF3 (S97) or total IRF3 in lysates of NC- and LINC02605-silenced HEK293T cells upon SeV infection for the indicated hours. **(E)** Western blot analysis of IRF3 expression in nuclear and cytoplasmic fractions in HEK293T cells transfected with NC or si#1 for 24h and then infected with SeV for the indicated hours, followed by nucleus-cytoplasm extraction. All data shown are from one representative experiment of at least three independent repeats with similar results.

**Figure 6:**
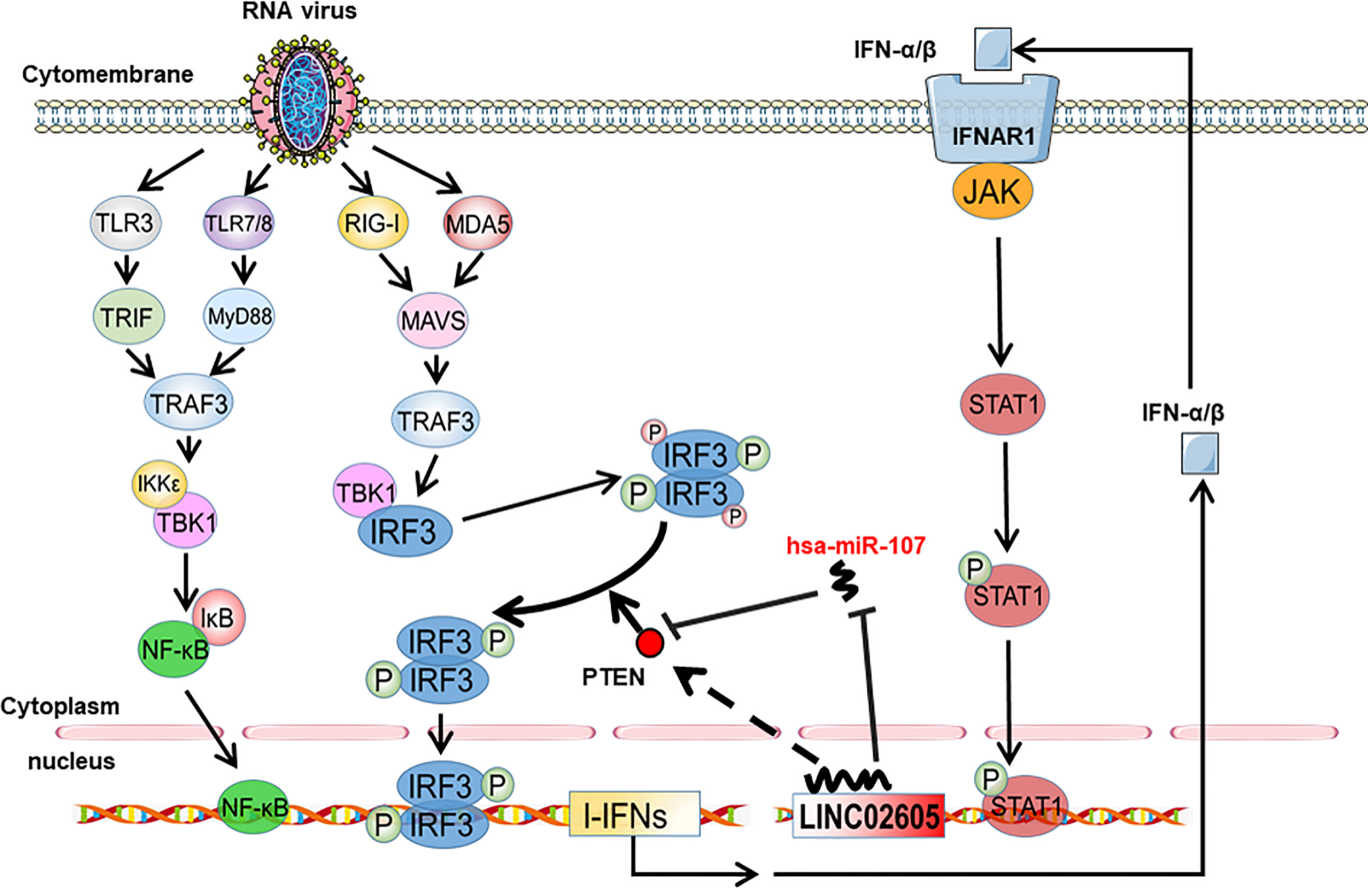
Working model of type I interferon-inducible LINC02605 in innate immunity. It promotes type I interferon production in antiviral immunity by binding hsa-miR-107 to strengthen IRF3 signaling in a positive feedback way.

PTEN was previously reported to positively regulate antiviral innate immunity. The phosphorylation status of IRF3 at Ser97 is critical for the retention in the cytosol. PTEN dephosphorylates IRF3 at Ser97 and promotes the nuclear translocation of IRF3, then leads to enhanced type I IFNs signaling (20). Because miR-107 and LINC02605 can regulate the expression level of PTEN, we then tried to monitor the effects of miR-107 or LINC02605 on the phosphorylation status of IRF3 at Ser97 and the nuclear translocation. Upon viral infection, hsa-miR-107 mimics can promote p-IRF3 (S97), while miR-107 inhibitor has the opposite effects, suppress p-IRF3 (S97) (**Figures 5A, B**). Additionally, overexpression of LINC02605 enhanced the expression of PTEN and reduced p-IRF3 (S97) level (**Figure 5C**). Conversely, knockdown of LINC02605 dampened the expression of PTEN and enhanced p-IRF3 (S97) level (**Figure 5D**) and led to reduced nuclear translocation of IRF3 (**Figure 5E**). Therefore, by controlling the nuclear localization of IRF3, LINC02605 positively modulates antiviral innate immunity (**Figure 6**).

## Discussion

The innate immune response constitutes the first line against virus invasion. RNA viral infection induces the changes of expression pattern of many responsive genes to guarantee proper strength of antiviral immunity is elicited. LncRNAs are non-coding RNAs with lengths greater than 200 nucleotides, which are involved in the regulation of many biological functions, including cell cycle regulation, cell differentiation, and immune response (25). The expression of lncRNAs can be regulated in time or spatial manner upon different stimulus. In order to gain comprehensive understandings of lncRNAs that may be involved in innate immune regulation, Arraystar LncRNA microarrays were used to screen the expression profiles of lncRNAs in HEK293T cells pre- and post- Sendai viral infection. Among the differential expressed lncRNAs upon viral infection, LINC02605 is up-regulated manifestly and is a potential regulator in antiviral immune response (21). In this study, we systemically studied the expression, functions, mechanism of actions of LINC02605 in antiviral immune response.

LINC02605 can be up-regulated by RNA/DNA virus and type I IFN. Analysis of transcription factors binding sites in the promoter region and signaling pathway inhibition assays revealed that the up-regulation of LINC02605 expression was dependent on the NF-κB and the Jak-STAT signaling pathway. The exact mechanism responsible for the transcriptional up-regulation of LINC02605 needs further studies. Upon viral infection, some lncRNAs are up-regulated or down-regulated to play a positive or negative feedback in the antiviral immune response. For instance, lnc-ISG20 is highly expressed after Influenza A virus (IAV) infection. It can bind to miR-326 to remove its inhibition on ISGs, thus inhibiting viral replication (26). And for another example, the differential expression of lnczc3h7a after virus infection is not reported. However, virus infection can promote the interaction of lnczc3h7a and TRIM25, thus augmenting RIG-I mediated antiviral immune response (14). Although the differentially expressed lncRNAs may be important mediators in innate immune response. There may exit some important lncRNA regulators whose expression levels are not changed after virus infection. The virus stimulation may change the conformation or the RNA binding partners of lncRNAs.

It is suggestive that LINC0605 may be a candidate regulator in innate immune response. By overexpression or knockdown experiments, we demonstrated that LINC02605 could promote RNA virus-induced type I interferon production, enhance activation of the ISRE promoter and inhibit viral replication. It is a positive player in antiviral immune response. Mechanistically, we found that LINC02605 could release the inhibition of expression of phosphatase and tensin homolog (PTEN) by binding hsa-miR-107. Notably, hsa-miR-107 not only inhibits PTEN’s expression but also negatively regulates the innate antiviral immune response. After in-depth analysis, we found that LINC02605 can regulate the phosphorylation level of Serine97 of interferon regulatory factor 3 (IRF3) by modulating PTEN levels, thus altering the nuclear translocation of IRF3. Nucleo-cytoplasmic isolation experiments showed that LINC02605 facilitates the nuclear-translocation of IRF3, playing a positive role in the regulation of innate immunity against viral invasion. In this study, we found that LINC02605 regulates the expression of PTEN through hsa-miR-107 in the antiviral immune system, which is consistent with a previous study in bladder cancer. Both RP11-79H23.3 (i.e. LINC02605 alias) and PTEN were significantly down-regulated in bladder cancer cells. RP11-79H23.3 can increase the expression of PTEN gene through absorption of hsa-miR-107. This leads to inhibition of PI3K/Akt signaling pathway, thereby inhibiting the development of bladder cancer (24).

IRF3 is the key transcription factor in innate immune response downstream TLR, RIG-I, STING and ZBP1 signaling *etc*. (15). The IRF3 activity can be delicately modulated by its phosphorylation level, protein stability, DNA binding activity *etc*. (27). For example, TRIM26 negatively regulates innate immunity by promoting the K48-linked ubiquitination and degradation of IRF3 (28). NSD3 methyltransferase mediates the methylation of IRF3 and positively regulates innate immune response (29). Lysine acetyltransferase 8 (KAT8) promotes IRF3 acetylation and inhibits innate immunity (30). Besides, IRF3 activation is also targeted by viruses to mediate immune evasion. For example, SARS-CoV M protein inhibits the IRF3 phosphorylation, whereas, N protein suppresses the transcriptional activity of IRF3 (31, 32). Our study found that LINC02605 is also an important regulator in the regulation of IRF3 activity. In view of the important roles of IRF3 in the downstream of TLR, RLR and cGAS-STING signaling pathway, it is possible that LINC02605 is also involved in the regulation of anti-bacterial or anti-DNA virus signaling. Until now, our study is the first report to link lncRNA with the expression of PTEN and the nuclear-translocation of IRF3 in the regulation of innate antiviral immunity, suggesting the complexity of IRF3 regulation and host innate immune regulation.

LncRNAs canonically interact with miRNAs to exert their functions (33). In analyzing the mechanism of action of LINC02605 in the process of antiviral infection, we constructed a bioinformatics network of lncRNAs-micro RNAs interactions, and predicted many micro RNAs with binding potential to LINC02605 by miTG score. In addition to hsa-miR-107, higher scoring micro RNAs included hsa-miR-182-5p, hsa-miR-511-5p, hsa-miR-96-5p, hsa-miR-222-5p and hsa-miR-548b-3p, *etc*. These micro RNAs may also be involved in the regulation of immunity to viral infection. It was reported that hsa-miR-182-5p expression was significantly upregulated in dendritic cells infected with human metapneumovirus (34). Activation of the Tat gene of HIV-1 virus promotes massive replication of HIV-1, and this process significantly upregulates intracellular hsa-miR-222-5p, which helps protect HIV-1-infected CD4^+^ T cells from apoptosis (35).

Several previous studies have suggested that hsa-miR-107 can be used as a potential biomarker for a variety of cancers. For example, in human squamous cell carcinoma, the expression of hsa-miR-107 is higher than that in non-tumor adjacent tissues, and its high expression is associated with the loss of PTEN expression and the deterioration of the disease progression (36). However, whether hsa-miR-107 plays a regulatory role in antiviral immunity has not been reported. In this work, we studied the relevant functions of hsa-miR-107 in the antiviral innate immune response and found that miR-107 plays a negative role in this process. In addition to PTEN as one of its targets, it worth further studies whether other targets of miR-107 are also involved in antiviral innate immune regulation.

LncRNAs may perform biological functions through a variety of mechanisms except for acting as ceRNAs. LncRNAs can regulate immune responses and inflammation by binding to proteins. As an example, long coding RNA nuclear paraspeckle assembly transcript 1 (NEAT1), the core structural component of the nuclear body paraspeckle is differentially expressed in the plasma of breast cancer patients, which promotes the migration and invasion of breast cancer cells by binding with hsa-miR-133b (37). At the same time, NEAT1, which is induced by estrogen in breast cancer, can form FOXN3-NEAT1-SIN3A inhibitory factor, through the interaction with FOXN3 and SIN3A protein complex, and promote epithelial-mesenchymal transformation (EMT) and metastasis, diffusion and invasion of breast cancer cells. High levels of NEAT1 are strongly associated with poor prognosis (38). It is also important to resolve the binding ability of LINC02605 to related RBPs. We used the catRAPID website to predict proteins that might interact with LINC02605. Based on the discriminative power (DP) and interaction strength scores between LINC02605 and the corresponding RBPs, the RBPs with the highest scores include SFPQ, SRSF10, ELAVL1 and others. In future studies, we will verify the interaction between candidate RBPs and lncRNA by RIP and RNA-pull down experiments. These studies may provide alternative mechanisms for LINC02605 in innate immunity. In the course of this project, a study reported that LINC02605’s neighboring Antisense IL-7 is induced by lipopolysaccharide (LPS) and interacts with nuclear protein p300 to mediate histone acetylation and chromatin remodeling to promote the transcription of inflammatory genes (39). It is interesting to test whether LINC02605 can also bind to p300 to function in the nucleus.

As mentioned earlier, LINC02605 is mainly located in the nucleus upon viral infection. How does a nuclear lncRNA function as a ceRNA? In published literature, some nuclear lncRNAs are reported to sponge microRNAs. For example, l lncRNA NEAT1, it can act as a sponge for miRNA such as hsa-miR-133b and miR-128-3p (40). Metastasis associated lung adenocarcinoma transcript 1 (MALAT1), which belongs to the same family as NEAT1, is mainly located in the nucleus after transcription, and hardly distributed in cytoplasm (41). In recent years, it has been reported that MALAT1 can promote the invasive lung cancer by interacting with hsa-miR-205 (42). There are also other evidences suggesting that nuclear lncRNAs can function as ceRNAs in regulating the transcription of related genes (43). Why nuclear lncRNAs can play the role of ceRNAs is still not clear at present. One possible explanation is that nuclear lncRNAs are cleaved into a small spliceosome by RNA enzymes and transported to the cytoplasm. Cytoplasmic localization confers this segment a spatial advantage as a signal transduction factor, enabling it to participate in intra- and inter-cellular signaling transduction (44). Specifically, by which way LINC02605 translocates to the cytoplasm and exerted its ceRNA function, in-depth research is needed in the future.

In summary, in this study, we identified LINC02605 as a novel positive regulator in antiviral innate immunity by regulating the expression level of PTEN and the nuclear translocation of IRF3. It extends the understanding of the regulation of innate immunity and offers new possible strategies for the intervention for related diseases.

## Data Availability Statement

The raw data supporting the conclusions of this article will be made available by the authors, without undue reservation.

## Author Contributions

RX performed experiments and wrote the draft manuscript. S-SY, R-RY, R-CT, J-WL, and XP performed experiments or analyzed data. JZ designed the project, wrote and revised the manuscript. All authors contributed to the article and approved the submitted version.

## Funding

This work was supported by grants from the National Natural Science Foundation of China (81873871 and 82071786), the interdisciplinary medicine Seed Fund of Peking University (BMU2021MX020) and the Fundamental Research Funds for the Central Universities.

## Conflict of Interest

The authors declare that the research was conducted in the absence of any commercial or financial relationships that could be construed as a potential conflict of interest.

## Publisher’s Note

All claims expressed in this article are solely those of the authors and do not necessarily represent those of their affiliated organizations, or those of the publisher, the editors and the reviewers. Any product that may be evaluated in this article, or claim that may be made by its manufacturer, is not guaranteed or endorsed by the publisher.
