# Supplementary material for: Interferon-Inducible LINC02605 Promotes Antiviral Innate Responses by Strengthening IRF3 Nuclear Translocation
Source: Front Immunol. 2021 Nov 5;12:755512. doi: 10.3389/fimmu.2021.755512 (PMC8602795; doi:10.3389/fimmu.2021.755512)
Supplement: Supplementary file 1 [file DataSheet_1.docx]

**Supplementary information, Fig. S1**


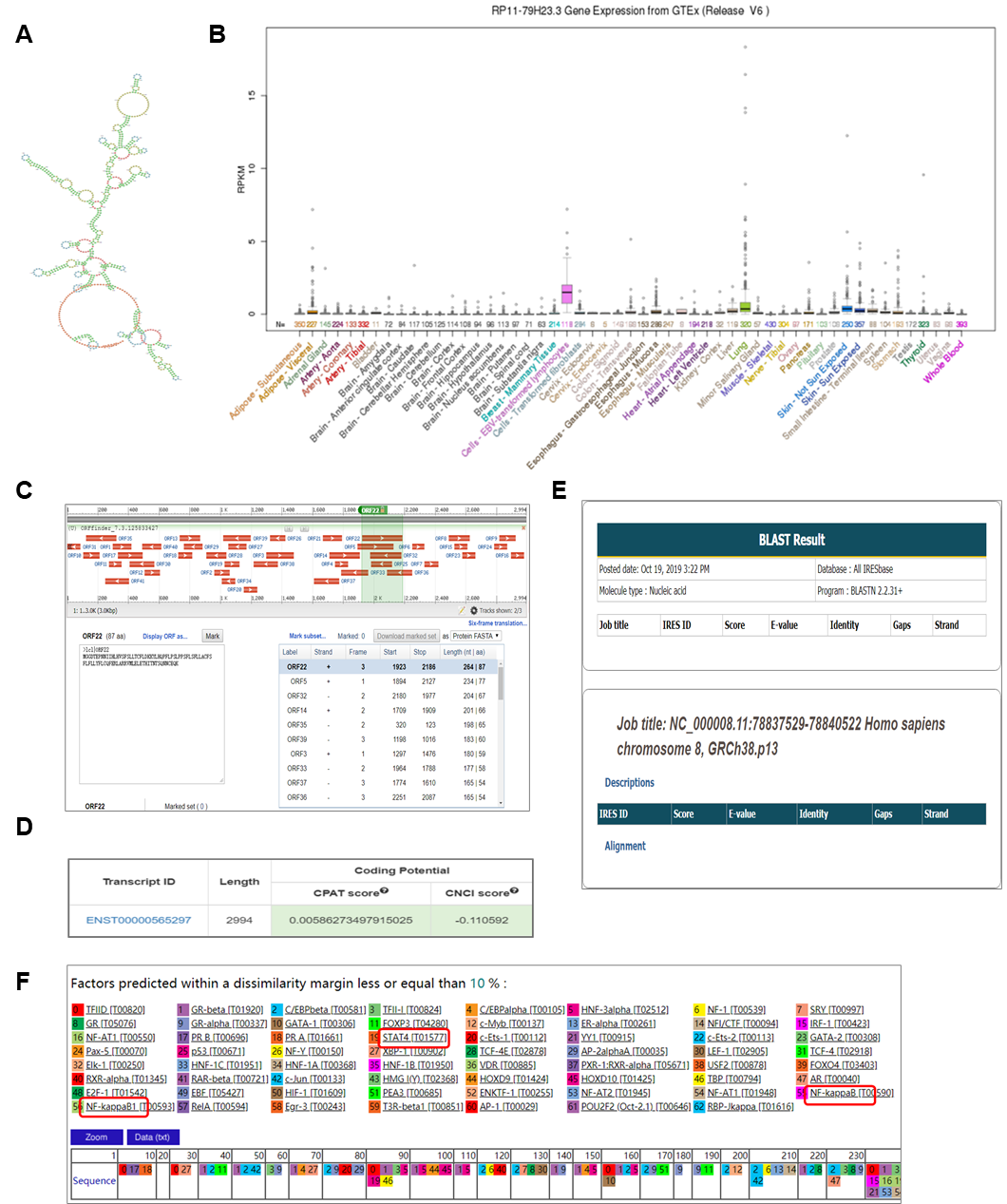


**Supplementary information, Fig. S2**


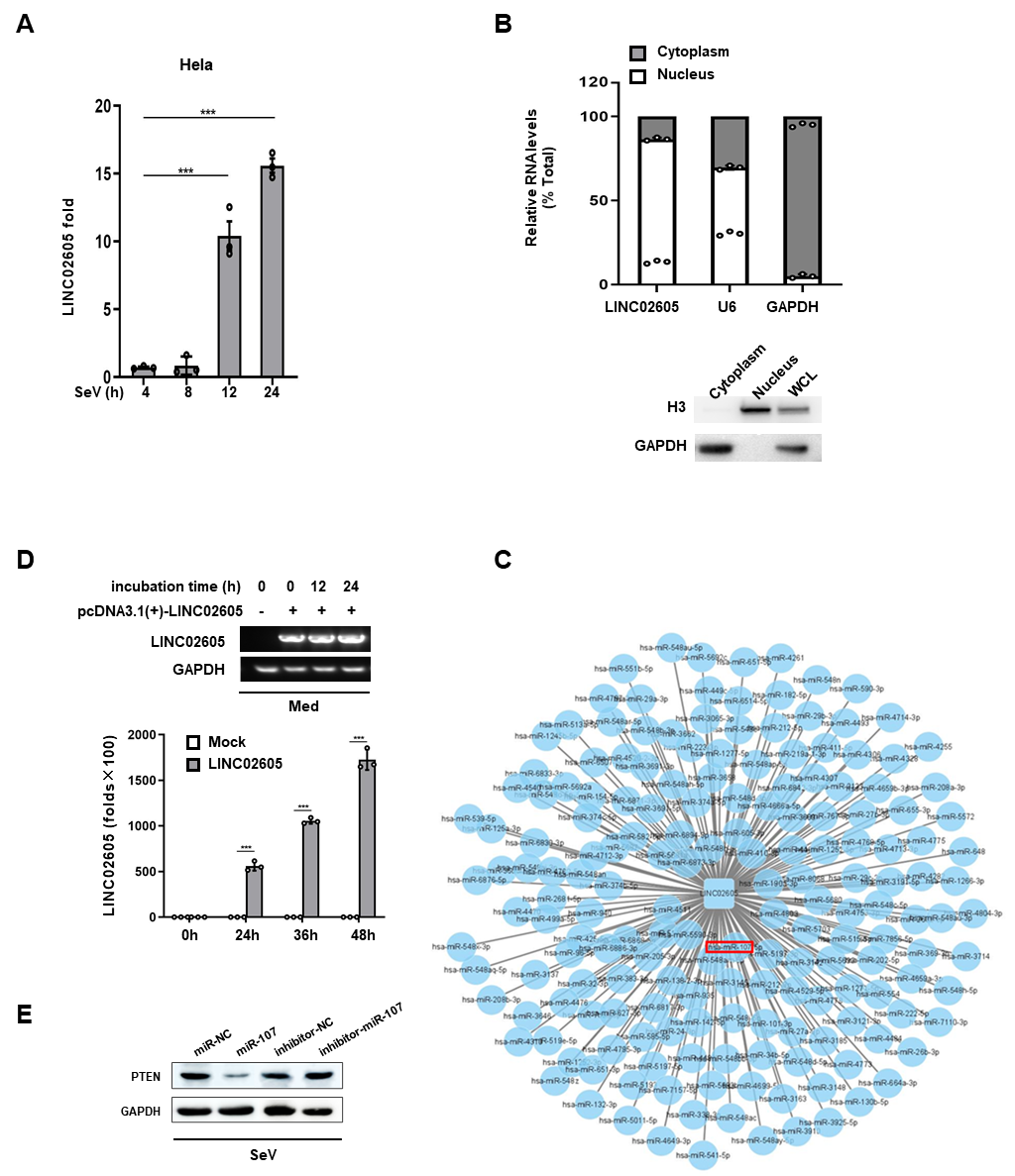


**Supplementary Figure legends**

**Supplementary Information, Figure. S1**  **(a)** The structure visualization of LINC02605 (<http://www.noncode.org/index.php>). (b) LINC02605 expression level in GETx (Release V6). The box plot shows that LINC02605 is highest expressed in EBV transformed lymphocytes. **(c, d)** The protein coding potential prediction of LINC02605 by using ORFfinder (<https://www.ncbi.nlm.nih.gov/orffinder>) and lnCAR (<https://lncar.renlab.org/explorer>). **(e)** IRES regions were not found in the full length sequence of LINC02605 by using the IRESbase (http://reprod.njmu.edu.cn/cgi-bin/iresbase/index.php). **(f)** Prediction of transcription factor binding sites analyzed by PROMO (<http://alggen.lsi.upc.es/cgi-bin/promo_v3>) in the promoter region ( ~ -2000 bp ~ +100 bp from TSS, transcription start site) of LINC02605.

**Supplementary Information, Figure. S2 (a)** Q-PCR analysis of LINC02605 expression in Hela cells infected with SeV for the indicated hours. **(b)** Q-PCR analysis (above) of the distribution of different RNAs following cytoplasm / nucleus fractionation of HEK293T cells infected with SeV for 12h. Western blot analysis (below) of cytoplasm / nucleus fractionation. **(c)** The lncRNA-miRNA competing endogenous RNA network. All the potentially micro RNAs interacting with LINC02605 were enriched by Diana-LncBase (http://carolina.imis.athena-innovation.gr/diana_tools/web), and the miRNAs were scored by miRBase database (<http://www.mirbase.org/index.shtml>). (**d**) Basic PCR (above) and RT-qPCR (below) analysis of LINC02605 expression efficiency of plasmid. **(e)** Western blot analysis of PTEN protein in hsa-miR-107 mimics or inhibitor of miR-107 transfected HEK293T cells infected with SeV for 12 hours.
